# *MIR2111-5* locus and shoot-accumulated mature miR2111 systemically enhance nodulation depending on HAR1 in *Lotus japonicus*

**DOI:** 10.1038/s41467-020-19037-9

**Published:** 2020-10-15

**Authors:** Nao Okuma, Takashi Soyano, Takuya Suzaki, Masayoshi Kawaguchi

**Affiliations:** 1 Division of Symbiotic Systems, National Institute for Basic Biology, 38 Nishigonaka, Myodaiji, Okazaki, Aichi 444-8585 Japan; 2 School of Life Science, SOKENDAI (The Graduate University for Advanced Studies), 38 Nishigonaka, Myodaiji, Okazaki, Aichi 444-8585 Japan; 3 Graduate School of Life and Environmental Sciences, University of Tsukuba, 1-1-1 Ten-noudai, Tsukuba, Ibaraki 305-8572 Japan

**Keywords:** Plant signalling, Rhizobial symbiosis

## Abstract

Legumes utilize a shoot-mediated signaling system to maintain a mutualistic relationship with nitrogen-fixing bacteria in root nodules. In *Lotus japonicus*, shoot-to-root transfer of microRNA miR2111 that targets *TOO MUCH LOVE*, a nodulation suppressor in roots, has been proposed to explain the mechanism underlying nodulation control from shoots. However, the role of shoot-accumulating miR2111s for the systemic regulation of nodulation was not clearly shown. Here, we find *L. japonicus* has seven miR2111 loci, including those mapped through RNA-seq. *MIR2111-5* expression in leaves is the highest among miR2111 loci and repressed after rhizobial infection depending on a shoot-acting HYPERNODULATION ABERRANT ROOT FORMATION1 (HAR1) receptor. *MIR2111-5* knockout mutants show significantly decreased nodule numbers and miR2111 levels. Furthermore, grafting experiments using transformants demonstrate scions with altered miR2111 levels influence nodule numbers in rootstocks in a dose-dependent manner. Therefore, miR2111 accumulation in leaves through *MIR2111-5* expression is required for HAR1-dependent systemic optimization of nodule number.

## Abstract (web summary)

In legumes, shoot-derived signals regulate rhizobial symbiosis to balance the benefits of N_2_ fixation with the energy costs of nodule production. Here Okuma *et al*. show that *Lotus* miR2111 is produced in the leaves from the *MIR2111-5* locus and is a graft-transmissable signal that enhances nodulation in the roots.

## Introduction

Land plants ensure their continuous growth under fluctuating conditions by sharing environmental information between the leaves and roots. For example, information about light conditions for the leaves^1^ and nitrogen (N) deficiency in the roots^2^ is transferred using a long-distance communication system^3^. These shoot–root communications are accomplished delivering signaling molecules between shoot and roots, such as proteins^1^, peptides^2,4–6^, phytohormones^7–9^, and RNA species^10–12^, which are produced locally, in response to various environmental cues. In *Arabidopsis*, HY5, a bZIP transcription factor, translocates from shoots to roots in response to light and regulates root growth and nitrate uptake^1^. C-TERMINALLY ENCODED PEPTIDE (CEP) family peptides produced in nitrate-deficient roots serve as N-demand signals and systemically regulate nitrate uptake through shoot-acting receptors, CEPRs, and shoot-to-root mobile CEPD peptides^2,4,6^.

Leguminous plants have evolved shoot-mediated long-distance signaling systems to maintain mutualism with rhizobia, soil N_2_-fixing symbiotic bacteria, that inhabit specialized symbiotic organs, root nodules^13–17^. Through this symbiotic interaction, rhizobia fix atmospheric N_2_ into ammonium, a plant-available form of N, in exchange for photosynthate from the host plant. Therefore, root nodule symbiosis is beneficial to host plants under N-limiting conditions. However, excessive nodule formation strongly inhibits host growth because N_2_-fixation is a highly energy-consuming process. To optimize the number of nodules, legumes utilize a long-distance negative-feedback mechanism known as autoregulation of nodulation (AON)^17^.

AON is achieved by signal transduction between roots and shoots through a shoot-acting leucine-rich repeat receptor-like kinase (LRR-RLK). The LRR-RLK was isolated from hypernodulation mutants of various legume species^13,18^, and designated HYPERNODULATION ABERRANT ROOT1 (HAR1) in *Lotus japonicus*^14–16^, SUPER NUMERIC NODULES (SUNN) in *Medicago truncatula*^19^, NODULE AUTOREGULATION RECEPTOR KINASE in soybean^20^, and SYM29 in pea^16^. Reciprocal grafting of these mutants demonstrated that these LRR-RLKs act in the shoot systemically to inhibit nodule formation in roots. These LRR-RLKs have an indispensable role in AON to perceive root-derived mobile CLV3/ESR-related (CLE) peptides, such as CLE ROOT SIGNAL 1, 2, and 3 (CLE-RS1, 2, and 3) in *L. japonicus*^5,21,22^, CLE12 and CLE13 in *M. trunctula*^23^, and RHIZOBIA-INDUCED CLE1 (RIC1) and RIC2 in soybean^24,25^. All of these peptides are synthesized in roots in response to rhizobial infection or high soil nitrate concentrations. In *L. japonicus*, tri-arabinosylation of CLE-RS2 peptide is required for the direct binding of HAR1 and suppression of nodulation in a HAR1-dependent manner^5^. Moreover, the inhibitory effects of CLE peptides on nodulation in *L. japonicus* depend partially on the gene known as *PLENTY*, which encodes hydroxyproline‐O‐arabinosyl transferase (HPAT)^26^. In *M. truncatula*, mutants of *ROOT DETERMINED NODULATION1*^27,28^, an orthologous gene to *PLENTY*, show a similar phenotype to *plenty*. Thus, arabinosylation of CLE peptides by HPATs is likely to be required in order for them to be recognized by LRR-RLKs. After CLE peptides are perceived by the shoot-acting LRR-RLK, nodulation in roots becomes inhibited by TOO MUCH LOVE (TML), the gene for which was isolated from hypernodulation mutants of *L. japonicus*^29,30^. Since *TML* encodes an F-box/kelch-repeat protein, it is hypothesized that TML may be involved in ubiquitin proteasome-mediated degradation of unknown target proteins that are associated with early nodule development^30^. *M. truncatula* has two *TML* orthologues, *TML1* and *TML2*, that inhibit nodule formation downstream of SUNN^31^. Hence, these factors that are involved in systemic signaling are well-conserved in legume species.

Two types of molecules generated in shoots downstream of LRR-RLK that are involved in AON have been reported to date. One is a phytohormone, cytokinin (CK). In *L. japonicus*, CK accumulates in shoots in a *HAR1*-dependent manner after inoculation with rhizobia^7^. When exogenously applied to shoots, CK causes a decrease in nodule numbers in a *TML*-dependent manner. The other factor is microRNA (miRNA) miR2111, which targets *TML* mRNA and enhances nodulation^12,32^. In *L. japonicus* and *M. truncatula*, the accumulation of mature miR2111s is drastically reduced in both shoot and roots in response to rhizobial infection and nitrate treatments, depending on *HAR1/SUNN*. Thus, *HAR1/SUNN* negatively regulates the accumulation of miR2111s, which target *TML* mRNA to enhance nodulation under non-inoculated or nitrate-deficient conditions^12,32^.

A substantial portion of miRNA gene families in plants consists of multiple loci^33–35^. Each individual locus in a certain miRNA gene family often exhibits different expression levels and patterns and sometimes is involved in distinct developmental processes even though the target genes are shared in the gene family^36–39^. Three loci for miR2111 (*MIR2111*-*1, -2*, and *-3*) have been reported in *L. japonicus*. Ectopic overexpression of *MIR2111-3* in hairy roots increases the amount of mature miR2111 and nodule numbers. Because the activity of *MIR2111-3* promoter is detected predominantly in the phloem of leaves, translocation of shoot-derived mature miR2111s into roots has been proposed to explain anticorrelation between the levels of mature miR2111s and of *TML* mRNA in roots. However, it remains unclear whether or not *MIR2111-3* is a responsible locus for AON since *MIR2111-3* expression levels and its dependency on HAR1 have not been evaluated. Moreover, systemic regulation by shoot-accumulating mature miR2111s of nodulation has not been clearly shown.

In this study, we identified four *L. japonicus* miR2111 loci, the expression of which was detected in leaves and controlled by HAR1 LRR-RLK. We attempted reverse genetic analyses such as CRISPR and short tandem target mimic (STTM) technologies to elucidate the function of highly regulated miR2111 loci on shoot-mediated control of nodulation. Furthermore, we examined whether or not shoot-derived miR2111 is sufficient for the systemic regulation of nodulation in roots by grafting experiments using miR2111 overexpressed line and downregulated lines.

## Results

### Identification of *MIR2111-2*, *-4*, *-5*, and *-7*, the expression of which is repressed by rhizobial infection in a *HAR1*-dependent manner

Three genomic loci for miR2111 precursors (*MIR2111*-*1, -2*, and *-3*) have been reported in *L. japonicus* (Supplementary Table 1). These miR2111 gene loci contain a total of five miR2111 hairpin sequences, which generate three mature miR2111 isoforms, miR2111a, miR2111b, and miR2111c^12^. We investigated additional potential miR2111 hairpin sequences using BLAST search with an improved reference genome assembly for *L. japonicus*^40^ (Gifu v1.2), and hairpin structure prediction^41,42^. In addition to the five known miR2111 hairpin sequences, we identified five sequences that are potentially processed to become miR2111s and designated a total of 10 miR2111 hairpin sequences as miR2111-A to miR2111-J (Supplementary Table 1 and Supplementary Fig. 1a). The five new sequences were predicted to form hairpin structures containing mature miR2111 sequences in their stems (Supplementary Fig. 1b). However, the predicted structures of three hairpin sequences (miR2111-F, -H, and -J) were not typical among reported miRNA structures since they have the miR2111 duplex at the proximal end of the hairpin structure^43–45^ (Supplementary Fig. 1b).

**Fig. 1:**
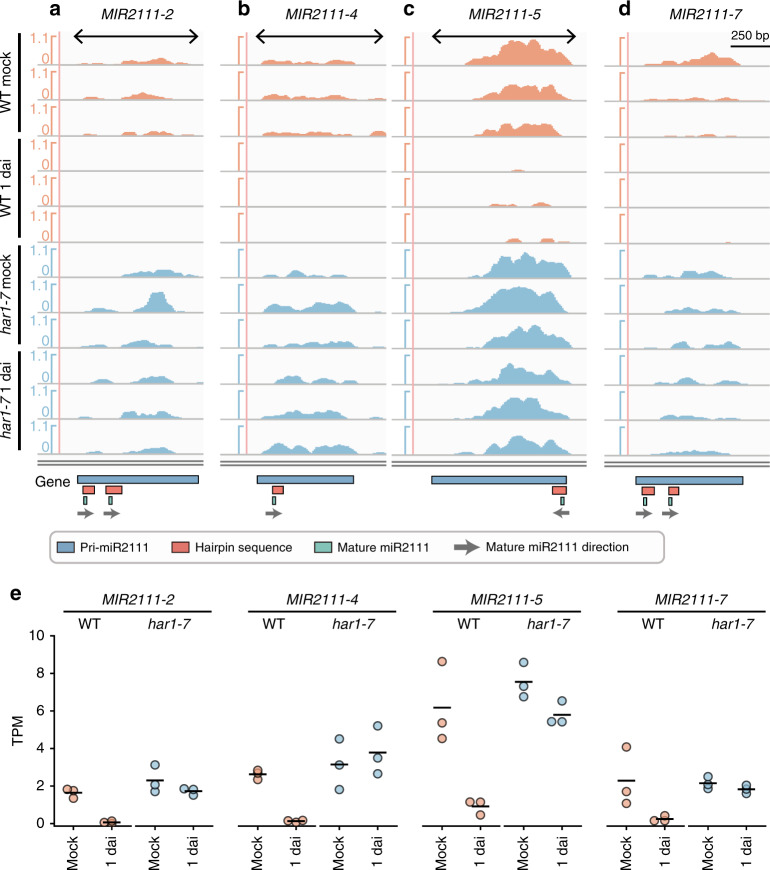
Expression of four *L. japonicus* miR2111 genes, including those identified by RNA-seq analysis. **a**–**d** RNA-seq read coverage of *MIR2111-2* (**a**), *MIR2111-4* (**b**), *MIR2111-5* (**c**), and *MIR2111-7* (**d**) were visualized by the Integrative Genomics Viewer. All data were acquired by RNA-seq of mature leaves of wild-type (MG-20) and *har1-7* plants that were inoculated with *M. loti* (1 day after inoculation) or mock-treated (control). RNA-seq libraries were prepared using poly(A) enrichment method, and miR2111 genes were predicted by RNA-Seq-based gene prediction using Stringtie version 1.3.4d with default settings. Read abundance normalized in bins per million (BPM) is shown. Gray arrows in the gene column represent the directions of mature miR2111 (*Bottom*). Regions used for the over-expression assay are indicated as black two-directional arrows (*Top*) (see Fig. 2). **e** Transcripts per million (TPM) values of four miR2111 genes. Scatterplots show individual biological replicates as dots. Bars indicate mean values.

Next, we carried out RNA-seq analyses in mature leaves of *L. japonicus* (2 weeks after germination) to examine the expression of miR2111 precursors and performed RNA-Seq-based gene prediction using Stringtie^46^ to identify functional miR2111 loci containing predicted hairpin structures (Fig. 1a–d), because miRNAs are generated by processing of primary transcripts of miRNA genes (pri-miRNAs), which are polycistronic in some cases. We detected four pri-miR2111 sequences and named their genes *MIR2111-2*, *MIR2111-4*, *MIR2111-5*, and *MIR2111-7* (Fig. 1a–d). *MIR2111-4* and *MIR2111-5* are monocistronic miR2111 loci containing miR2111-F and miR2111-G, respectively. *MIR2111-2 and MIR2111-7* are polycistronic and possesses dual-hairpin-structure sequences. *MIR2111-2* contains the sequences for miR2111-B and miR2111-C, whereas *MIR2111-7* possesses the sequences for miR2111-I and miR2111-J (Supplementary Fig. 1a, b and Supplementary Table 1). The nucleotide sequence of *MIR2111-7* showed 96% identity with that of *MIR2111-2*, suggesting that these two loci are paralogous. Furthermore, we searched new potential miR2111 genes that were not detected from our RNA-seq data by BLASTn search using all miR2111 gene sequences as the query. We found a *MIR2111-4* paralogous sequence and named it *MIR2111-6*, which showed 93% identity with *MIR2111-4* and contained the sequence for miR2111-H (Supplementary Fig. 1a).

Apart from *MIR2111-1* on chromosome 1, each monocistronic and polycistronic miR2111 locus was tandemly paired and arranged near the pericentromeric region of chromosome 3 (Supplementary Fig. 1a, b). It is noteworthy that three of the four pri-miR2111s that were expressed in leaves, *MIR2111-4*, *MIR2111-5*, and *MIR2111-7*, were different to known miR2111 loci. In addition, the *MIR2111-2* transcript detected in this study was much longer (789 bp) than that reported previously (312 bp)^12^.

Expression of *MIR2111-2*, *MIR2111-4*, *MIR2111-5*, and *MIR2111-7* was repressed in wild-type (WT) leaves in response to rhizobial inoculation but not in the *har1-7* mutant (Fig. 1a–d), suggesting that expression of these miR2111 genes were negatively regulated by *HAR1*. *MIR2111-5* showed threefold to fivefold higher transcripts per million (TPM) values than the other miR2111 genes expressed in leaves of mock-treated WT and *har1-7* (Fig. 1b). Expression of *MIR2111-1*, *MIR2111-3*, and *MIR2111-6* was below detectable levels in our RNA-seq data (Supplementary Fig. 2).

### *MIR2111-2 and MIR2111-5* promote nodulation and mature miR2111 accumulation

We evaluated nodulation phenotypes of *L. japonicus* roots ectopically overexpressing the miR2111 genes we identified to confirm whether or not the genes are functional. We chose *MIR2111-2*, *MIR2111-4*, and *MIR2111-5* from among the four miR2111 genes that demonstrated expression in leaves. *MIR2111-7* was not incorporated into the functional analyses since it was over 90% identical to *MIR2111-2*. For the overexpression assay, DNA fragments fully covering pri-miRNA regions (~700–900 bp) were expressed under an *L. japonicus Ubiquitin* promoter^47^ (Supplementary Fig. 1a–d). We generated hairy roots transformed with either *proUBQ:MIR2111-2*, *proUBQ:MIR2111-4*, or *proUBQ:MIR2111-5*. Nodule numbers were counted in transformed roots displaying fluorescence of a GFP transformation marker at 21 days after inoculation (dai) with DsRed-labeled *M. loti*. Furthermore, we conducted qRT-PCR analyses to examine the accumulation of mature miR2111s and *TML* mRNA in transformed roots at 5 dai.

**Fig. 2:**
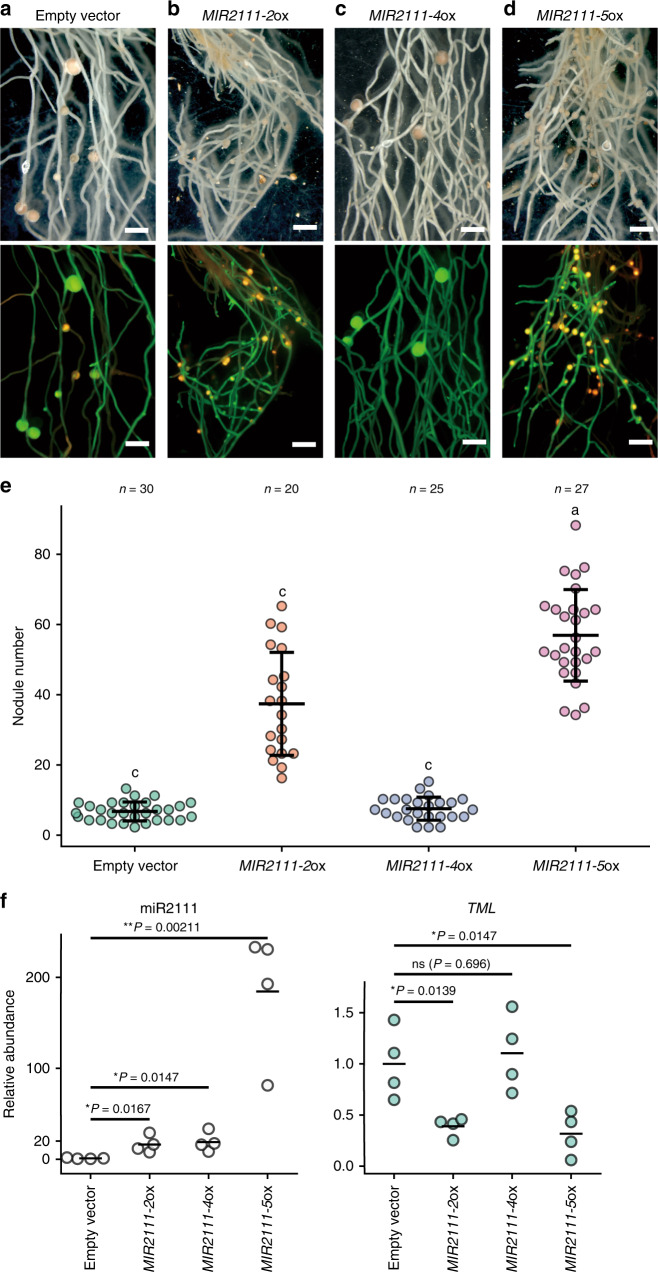
Overexpression of *MIR2111-2* and *MIR2111-5* in hairy roots promoted nodulation. **a**–**d** Nodulation at 21 days after inoculation (dai) on hairy roots transformed with an empty vector (**a**), *proUBQ:MIR2111-2* (**b**), *proUBQ:MIR2111-4* (**c**), and *proUBQ:MIR2111-5* (**d**). Bright images (upper) and corresponding fluorescence images as a GFP transformation marker and DsRed constitutively expressing in *M. loti* (lower). Scale bars: 2 mm. **e** Nodule numbers in hairy roots transformed with an empty vector, *proUBQ:MIR2111-2*, *proUBQ:MIR2111-4*, and *proUBQ1:MIR2111-5* (21 dai). Bars indicate mean ± standard deviation. Different letters indicate significant differences (*P* < 0.05) from Tukey’s honestly significant difference test. **f** qRT-PCR analyses of mature miR2111 and *TML* in transformed hairy roots (5 dai). *n* = 4 individual biological replicates for each treatment. Bars indicate mean values. Two-sided Student’s *t* test was used to determine statistical difference compared with empty vector control: *P < 0.05; ** *P* < 0.01; n.s. not significant. **e**, **f** Scatterplots show individual biological replicates as dots.

Nodule numbers were significantly increased in roots transformed with *proUBQ:MIR2111-2* and *proUBQ:MIR2111-5* compared with empty vector controls (Fig. 2a–e); the mean nodule numbers were about fivefold and eightfold higher in *proUBQ:MIR2111-2* and *proUBQ:MIR2111-5* roots, respectively (Fig. 2e). In addition, these transformed roots produced mature nodules that were apparently smaller than those in control roots, similarly to *tml* roots^29^ (Fig. 2a–d). *proUBQ:MIR2111-2* and *proUBQ:MIR2111-5* also increased the accumulation of mature miR2111s about 20-fold and 180-fold over controls, respectively (Fig. 2f). Conversely, the abundance of *TML* mRNA was decreased in these transformed roots (Fig. 2f). These results indicated that *MIR2111-2* and *MIR2111-5* enhanced nodulation and inhibited *TML* mRNA accumulation similarly to *MIR2111-3*^12^. Therefore, we concluded that *MIR2111-2* and *MIR2111-5* were functional in promoting nodule formation by influencing the accumulation of *TML* mRNA.

However, *proUBQ:MIR2111-4* influenced neither the number of nodules nor *TML* mRNA accumulation, even when the levels of mature miR2111 in the transformed roots we detected by qRT-PCR were comparable with those in roots transformed with *proUBQ:MIR2111-2* (Fig. 2f). This result suggested that *MIR2111-4* is not functional with respect to regulating nodule numbers.

### *MIR2111-5* is expressed predominantly in the phloem of mature leaves in a *HAR1-*dependent manner

Our RNA-seq analyses showed that, among *L. japonicus* miR2111 loci, *MIR2111-5* most abundantly accumulated in leaves of non-inoculated plants. In addition, compared with the other miR2111 loci tested, *MIR2111-5* overexpression in roots was the most effective at increasing the number of nodules. Therefore, we focused on *MIR2111-5* in further analyses.

**Fig. 3:**
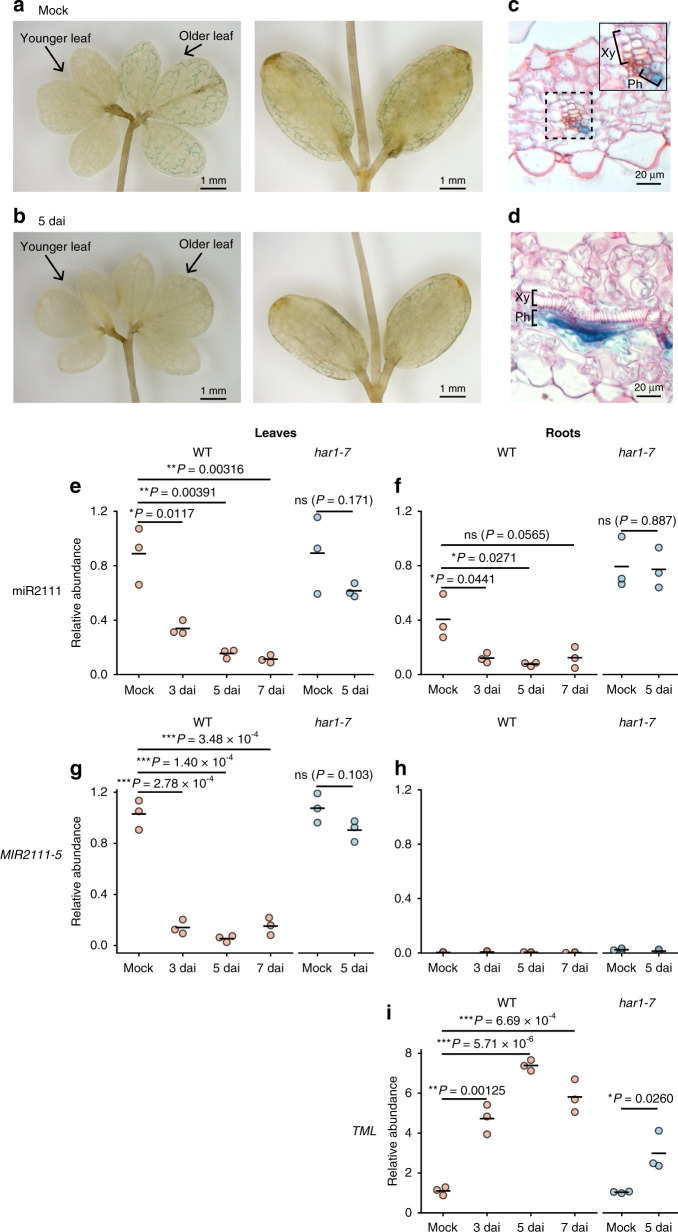
*MIR2111-5* was expressed mainly in the phloem of mature leaves. **a**–**d** GUS expression controlled by a 3.0-kb DNA fragment upstream of *MIR2111-5* in true leaves (left) and cotyledon (right) of plants mock-treated (**a**, **c**, **d**) and inoculated with *M. loti* (5 days after inoculation) (**b**) were incubated in GUS staining buffer for 3 h. **c**, **d** Leaf sections counterstained with 0.1% safranin for 10 min. Xy xylem, Ph phloem. **e**–**h** qRT-PCR analyses of mature miR2111s (**e**, **f**) and *MIR2111-5* (**f**, **g**), and *TML* (**i**) in leaves (**e**, **g**) and roots (**f**, **h**, **i**) at indicated days after inoculation (dai) with *M. loti*. Scatterplots show individual biological replicates as dots. Bars indicate mean values. All values were normalized by the mean value of wild-type mock-treated leaves. Two-sided Student’s *t* test was used to determine statistical difference compared with mock control: *P < 0.05; **P < 0.01; ***P < 0.001; n.s. not significant.

In order to characterize the spatial expression pattern of *MIR2111-5*, a 3.0-kb DNA fragment upstream of *MIR2111-5* was inserted upstream of a GUS reporter gene. Histochemical GUS staining assay was conducted using plants stably transformed with this reporter construct (*proMIR2111-5:GUS*). GUS staining was detected along with vascular bundles in true leaves and cotyledons of 2-week-old plants (Fig. 3a). At this point after germination, *L. japonicus* MG-20 seedlings have two sets of fully expanded leaves. GUS expression was detected strongly in the more mature leaves, while much weaker expression was seen in younger leaves (Fig. 3a). GUS expression tended to be clear in vascular bundles around the leaf margins and cotyledons, but was not detected in midveins (Fig. 3a). In contrast, GUS signals were negligible in stems, roots, and root nodules (Supplementary Fig. 3a–d). This GUS expression along with vasculatures was attenuated within 5 dai with *M. loti* (Fig. 3b). Cross-sections of leaves revealed that the GUS reporter was expressed mainly in the phloem (Fig. 3c, d), which is similar to the expression pattern previously reported for *MIR2111-3*^12^.

Next, we examined the effects of rhizobial infection on the accumulation of mature miR2111s, *MIR2111-5*, and *TML* mRNA in leaves and roots by qRT-PCR. Mature miR2111s were clearly detected in both leaves and roots of mock controls, and accumulation decreased within 3 dai in a *HAR1*-dependent manner (Fig. 3e, f). *TML* mRNA levels were negatively correlated with the accumulation of mature miR2111s in roots (Fig. 3i). Similar to mature miR2111s, the abundance of *MIR2111-5* in leaves also altered in response to inoculation with *M. loti* depending on *HAR1* (Fig. 3g). However, the accumulation of *MIR2111-5* in roots was sustained at very low levels or below detectable levels even in *har1-7* roots where mature miR2111s accumulated in abundance (Fig. 3h). Patterns of endogenous *MIR2111-5* accumulation were similar to those of GUS expression driven by its own promoter (Fig. 3a, b and Supplementary Fig. 3a–d).

### The accumulation of mature miR2111s in shoots is sufficient to enhance nodulation in roots

According to the current hypothesis, mature miR2111s are synthesized in leaves and translocated to roots via phloem vessels to activate nodulation through post-transcriptional inhibition of *TML* mRNA accumulation^12^. Consistent with this hypothesis, promoter activity of *MIR2111-5* was predominantly detected in the phloem of leaves and cotyledons (Fig. 3a–d). Indeed, *MIR2111-5* was abundant in leaves of uninoculated plants (Fig. 3g). Nevertheless, it was not clear whether shoot-derived miR2111 truly be able to systemically enhance nodulation in roots.

**Fig. 4:**
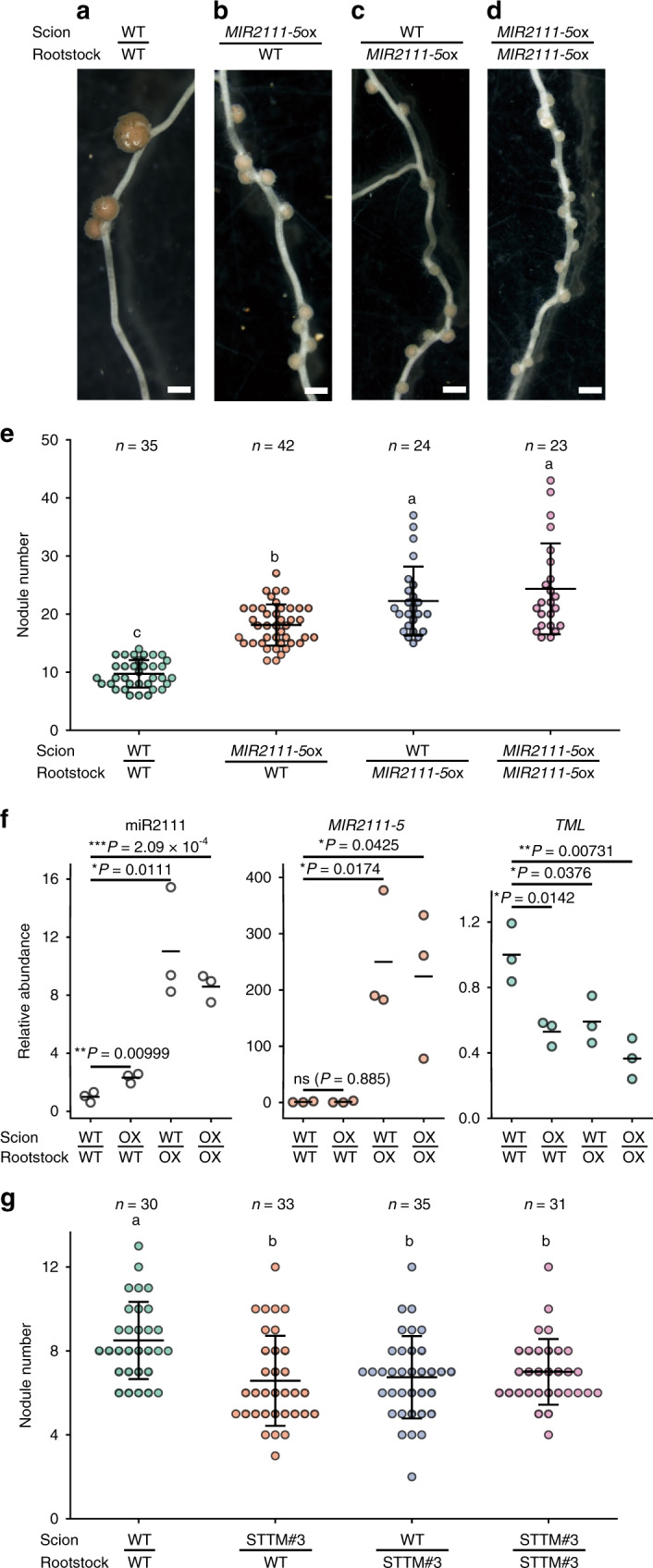
Reciprocal grafting between wild-type and either *MIR2111-5* overexpression or STTM2111 plants. **a**–**e** Nodulation at 28 days after inoculation (dai) in wild-type (**a**, **b**) and *MIR2111-5*ox (**c**, **d**) rootstocks grafted with wild-type (**a**, **c**) and *MIR2111-5*ox (**b**, **d**) scions. Scale bars: 1 mm. **e** Nodule numbers on rootstocks of grafted plants (28 dai). **f** qRT-PCR analyses of mature miR2111s, *MIR2111-5*, and *TML* in rootstocks of the grafted plants (5 dai). OX represents *MIR2111-5*ox plants. All values were normalized by the mean value of wild-type self-grafting plants. *n* = 3 individual biological replicates for each treatment. Bars indicate mean values. Two-sided Student’s *t* test was used to determine statistical difference compared with wild-type self-grafting plants: *P < 0.05; **P < 0.01; ***P < 0.001; n.s. not significant. **g** Nodule numbers in rootstocks of grafted plants between WT and STTM2111 (21 dai). STTM represents STTM2111 plants. **e**–**g** Scatterplots show individual biological replicates as dots. Different letters indicate significant differences (*P* < 0.05) from Tukey’s honestly significant difference test. **e**, **g** Bars indicate mean ± standard deviation.

To clarify the functional relevance of shoot-derived miR2111s in nodulation, we generated stable *proUBQ:MIR2111-5* transgenic lines (*MIR2111-5*ox) (Supplementary Fig. 4). *MIR2111-5*ox displayed significantly increased nodule and infection thread numbers, indicating *MIR2111-5* overexpressed lines successfully generated (Supplementary Fig. 4). Next, we performed reciprocal grafting between *MIR2111-5*ox and WT plant. Scions overexpressing *MIR2111-5* significantly increased nodule numbers in WT rootstocks (28 dai) (Fig. 4a–e). This result indicated that *MIR2111-5* overexpression in shoots was sufficient to promote nodulation. Next, we examined the effects of *MIR2111-5* overexpressed scions on the accumulation of mature miR2111s, *MIR2111-5*, and *TML* mRNA in rootstock using qRT-PCR. WT rootstocks grafted with *MIR2111-5*ox scions showed significant increases in mature miR2111s and a reduction of *TML* mRNA levels (5 dai) (Fig. 4f), indicating that *MIR2111-5* overexpression in shoots can systematically regulate the accumulation of mature miR2111s and *TML* mRNA in roots. Grafting between *MIR2111-5*ox scions and *tml-4* rootstocks or *har1-7* scions and *MIR2111-5*ox rootstocks did not exhibit additive effects on nodule numbers compared with self-grafted mutant plants, suggesting that *MIR2111-5* functioned in the same genetic pathway as *HAR1* and *TML* in the control of nodule number (Supplementary Fig. 5).

In contrast to mature miR2111s, *MIR2111-5* levels in WT rootstocks grafted with *MIR2111-5*ox scions were very low or below detectable levels (Fig. 4f). To confirm whether or not shoot-derived mature miR2111s promote nodulation in roots, we designed a short tandem target mimic (STTM) construct^48^ to prevent the accumulation of mature miR2111s (*proUBQ:STTM2111*) and generated stable *proUBQ:STTM2111* transgenic lines (STTM2111). In leaves of STTM2111, the abundance of mature miR2111s was reduced by less than half of that in WT plants, whereas the levels of *MIR2111-5* were not influenced, indicating that STTM2111 successfully inhibits mature miR2111s (Supplementary Fig. 6). Next, we carried out reciprocal grafting experiments between STTM2111 and WT plants. STTM2111 scions significantly reduced nodule numbers in the WT rootstocks to those equivalent to STTM2111 rootstocks (Fig. 4g). Thus, the amount of mature miR2111s generated in shoots is crucial to increasing nodule numbers in the roots.

### *MIR2111-5* locus is required to increase root nodule numbers

**Fig. 5:**
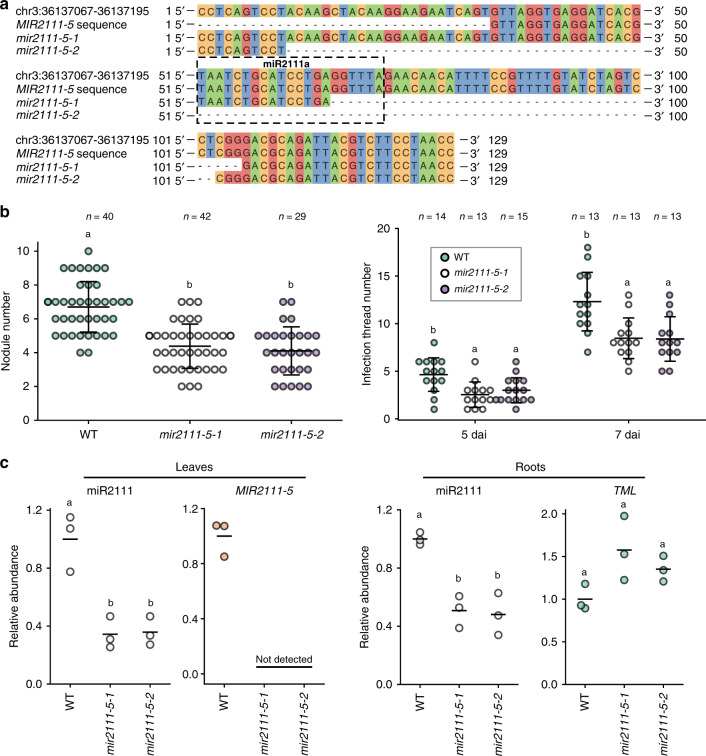
*MIR2111-5* knockout significantly inhibited nodulation and accumulation of mature miR2111s. **a** Nucleotide sequences of *MIR2111-5* and its knockout lines. **b** The numbers of nodules (21 days after inoculation) and infection threads (5 and 7 days after inoculation) on roots of WT, *mir2111-5-1*, and *mir2111-5-2*. Bars indicate mean ± standard deviation. **c** qRT-PCR analyses of mature miR2111s, *MIR2111-5*, and *TML* in leaves and roots at 14 and 7 days after germination, respectively. *n* = 3 individual biological replicates for each treatment. Bars indicate mean values. **b**, **c** Scatterplots show individual biological replicates as dots. Different letters indicate significant differences (*P* < 0.05) from Tukey’s honestly significant difference test.

Although overexpression of *MIR21111-5* significantly increased nodule numbers and mature miR2111 accumulation in roots, it remained unknown to what degree *MIR2111-5* locus contributes to producing mature miR2111s and controlling nodule numbers. To address this issue, we generated two knockout lines of *MIR2111-5*, *mir2111-5-1*, and *mir2111-5-2*, using a CRISPR technology with dual gRNA-containing constructs (Fig. 5a; see “Methods”). In *mir2111-5-1* and *mir2111-5-2*, the mature miR2111 sequence was partially or completely deleted (Fig. 5a).

The deletion of *MIR2111-5* significantly decreased the nodule and infection thread number compared with the WT (Fig. 5b). Furthermore, the abundance of mature miR2111s in these leaves and roots was reduced to less than half of that seen in the WT (Fig. 5c). *TML* mRNA levels were not significant but tended to be higher in roots of *mir2111-5* mutants than those of the WT (Fig. 5c). These results suggested that *MIR2111-5* locus is necessary to increase the amount of mature miR2111s in leaves and roots and the number of nodules in roots.

## Discussion

Land plants adapt to environmental stimuli, such as soil nutrient deprivation at the whole-plant level by shoot-mediated signaling communication via vascular bundles^3^. In this study, we investigated the functions of miR2111 genes in the shoot-mediated nodulation control system known as AON, focusing, in particular, on *MIR2111-5* locus. *L. japonicus* possesses at least seven miR2111 genes, including those we identified and four genes expressed in leaves (Fig. 1 and Supplementary Fig. 1a, b). Grafting experiments demonstrated that the accumulation of mature miR2111s in shoots was sufficient to increase root nodule numbers and reduce the accumulation of their targets, *TML* mRNA, in roots (Fig. 4). Moreover, we found that *MIR2111-5* locus, which was expressed mainly in leaves (Fig. 3 and Supplementary Fig. 3), was required for the regulation of nodule number and accumulation of mature miR2111s in roots (Fig. 5). Thus, mature miR2111s accumulation in leaves through *MIR2111-5* expression is necessary to systemically optimize nodule number in a *HAR1*-dependent manner.

Four miR2111 genes, *MIR2111-2*, *MIR2111-4*, *MIR2111-5*, and *MIR2111-7*, were expressed in leaves and repressed after rhizobial inoculation in a *HAR1*-dependent manner, whereas expression of the others was not detected by our RNA-seq analyses (Fig. 1 and Supplementary Fig. 2). All of these miR2111 genes have complementary sequences to *TML* mRNA. Therefore, it is assumed that miR2111 genes that were expressed in leaves have functional redundancies and potentially work in the AON pathway (Fig. 1). Indeed, overexpression of *MIR2111-5* and *MIR2111-2*, of which the sequence is 96% identical to that of *MIR2111-7*, significantly increased nodule numbers. In contrast, overexpression of *MIR2111-4* did not affect nodule numbers; nonetheless, mature miR2111s accumulated at high levels in transformed roots (Fig. 2). This may have been due to the atypical secondary structure of miR2111-F derived from *MIR2111-4* (Supplementary Fig. 1b). Unlike the hairpin structures of *MIR2111-2*^12^ and *MIR2111-5*, the mature miR2111 sequence in the predicted miR2111-F secondary structure is located at the 5′ end of its stem (Supplementary Fig. 1b). Nucleotide lengths proximal to the miRNA/miRNA* duplex affect the efficiency of correct miRNA processing in *Arabidopsis*^43–45^. Although we detected an increase of mature miR2111s in roots overexpressing *MIR2111-4*, miRNA generated from miR2111-F might not have nucleotide sequence patterns that are sufficient to target *TML* mRNA (Fig. 2). This notion is supported by the lack of a significant change in *TML* mRNA accumulation in roots overexpressing *MIR2111-4*. Although overexpression of *MIR2111-3* increased nodule numbers in the previous study^12^, expression of *MIR2111-3* was undetectable levels in our RNA-seq of *L. japonicus* leaves (Supplementary Fig. 2). *MIR2111-3* may be expressed under conditions different from those where the other four loci expressed.

It has been proposed that shoot-derived mature miR2111s are translocated to roots via the phloem to enhance nodulation^12^. However, it was unknown whether or not the accumulation of mature miR2111s in shoots leads to an increase in root nodule numbers. We carried out grafting experiments and demonstrated that WT rootstocks grafted with *MIR2111-5* overexpressed scions exhibited increased root nodule numbers and mature miR2111 levels and repressed *TML* mRNA levels (Fig. 4a–f). Since the levels of *MIR2111-5* were not increased in WT rootstocks, it is unlikely that the increment in mature miR2111s in rootstocks was due to the movement of *MIR2111-5* from shoots. These results indicated that *MIR2111-5* overexpression in the shoot was sufficient to systemically regulate root nodule numbers and *TML* mRNA levels. Furthermore, results from grafting between *MIR2111-5*ox and AON mutants, *har1-7* and *tml-4*, suggested that *MIR2111-5* functions in the same genetic pathway as these AON factors (Supplementary Fig. 5). In contrast to *MIR2111-5ox*, scions constitutively expressing *proUBQ*:*STTM2111* inhibited the accumulation of mature miR2111s (Supplementary Fig. 6) and decreased nodule numbers in WT rootstocks (Fig. 4f). Thus, the shoot-accumulating mature miR2111s influences root nodule numbers in a dose-dependent manner.

Among the seven miR2111 loci, *MIR2111-5* showed the highest level of TPM in *L. japonicus* leaves (Fig. 1). When overexpressing in roots, *MIR2111-5* was the most effective at increasing nodule numbers among miR2111 genes we examined (Fig. 2). These results implied that *MIR2111-5* might significantly contribute to the regulation of nodule numbers in *L. japonicus*. Knockout lines of *MIR2111-5* support this notion. Even though there are multiple loci for miR2111 genes in the *L. japonicus* genome (Fig. 1 and Supplementary Fig. 1a), as for other miRNA gene families in plants^33^, a single *mir2111-5* mutation decreased miR2111 levels in leaves and roots to less than half of WT (Fig. 5b). In addition, the *MIR2111-5* deletions resulted in the significant inhibition of nodule formation. As for *MIR2111-3* expression patterns^12^, *proMIR2111-5:GUS* expression was observed in the phloem of leaves (Fig. 3a, c, d) but not in stems, roots, or nodules (Supplementary Fig. 3). The accumulation of *MIR2111-5* was hardly detected in roots using qRT-PCR (Fig. 3h). Considering this expression pattern, it is likely that the decrease in nodule numbers and mature miR2111 levels in roots of *MIR2111-5* knockout lines was caused by the attenuation of mature miR2111 levels in leaves (Fig. 5c). The expression in the phloem, which is convenient to explain the shoot-to-root movement of mature miR2111s through the vasculature, was diminished after rhizobial inoculation (Fig. 3b). Thus, *MIR2111-5* accumulation in leaves is regulated at the transcriptional level. The decrease in *MIR2111-5* expression in leaves in response to rhizobial inoculation correlated well with the accumulation patterns of mature miR2111s in leaves and roots (Fig. 3). The HAR1-dependent transcriptional regulation of miR2111 genes including *MIR2111-5* in leaves would systemically influence nodule numbers in roots. Taken together with the results of grafting experiments, it is likely that mature miR2111s generated in shoots are translocated to roots and post-transcriptionally silence *TML* mRNA.

Phloem sap contains a defined subset of miRNA species, and some of these specifically accumulate under various nutrient-limiting conditions^49–51^. In *Arabidopsis* and tobacco, shoot-to-root mobile miR399s, which accumulate in shoots in response to the deprivation of inorganic phosphate (Pi), post-transcriptionally regulate mRNA for PHO2, which encodes ubiquitin-conjugating E2 enzyme 24^10,49^. Since PHO2 suppresses Pi uptake in roots, shoot-derived miR399s maintain Pi homeostasis through the regulation of their target mRNA levels. Interestingly, Pi-limitation induces the accumulation of mature miR2111 in rapeseed phloem sap^50,51^. *Arabidopsis* and tobacco miR2111s, which also accumulate in response to Pi-limitation^50,52^, are predicted to target mRNA for *TML* orthologues^52^. In legumes, the control of miR2111 production depends on nitrate availability^12,32^. Recently, it has been reported that in *M. truncatula*, nitrogen-deprivation in roots induces the production of mature miR2111s in shoots through CEP family peptide-mediated long-distance signaling, in order to enhance nodulation^32^. Although the biological functions of *TML* orthologues and roles of miR2111s in *Arabidopsis* and tobacco have not yet been identified, shoot-derived miRNA accumulation in roots may be a widespread mechanism of adaptation to nutritional changes in plants.

## Methods

### Plant materials and bacterial resources

We used *L. japonicus* accession MG-20^53^ as the WT. *har1-7*^29^ and *tml-4*^30^ mutants were derived from MG-20. *proMIR2111-5*:GUS transgenic lines, *MIR2111-5* overexpression lines, *proUBQ:STTM2111* transgenic lines, and *MIR2111-5* knockout lines were generated in the MG-20 background. *M. loti* MAFF303099 and MAFF303099 constitutively expressing dsRED were used for the *L. japonicus* inoculum.

### Plant culture conditions and bacterial inoculation

Sterilized *L. japonicus* seeds were germinated on 0.9% agar medium containing Broughton and Dilworth solution (B&D)^54^ without any nitrogen source for 3 days at 24 °C (16 h of light, 8 h of darkness).

For the nodulation and infection phenotyping assay, plants were transplanted on autoclaved vermiculite supplemented with B&D containing 0.5 mM KNO_3_ and inoculated with *M. loti* carrying dsRED. Plants were inoculated 3 days after being transplanted and harvested within indicated days after inoculation (dai) to analyze the nodulation and infection phenotypes.

For the RNA-seq, qPCR assay, and promoter GUS assay, plants were transplanted into autoclaved vermiculite containing B&D without a nitrogen source. Plants were inoculated either with or without *M. loti* carrying dsRED at indicated time points and harvested 14 days after germination.

For the grafting assay, plants were sown on vertical 0.9% agar plates for 2 days in darkness and 2 days under light/dark conditions. The 4-day-old seedlings were cut at the basal hypocotyls and scions were grafted on rootstocks, as described previously^29^. Grafted plants were grown horizontally on the sterilized water-absorbed filter paper for 1 week and then transplanted to autoclaved vermiculite containing B&D either with or without 0.5 mM KNO_3_ for the nodulation assay and qRT-PCR assay, respectively. Seven days after being transplanted, grafted plants were inoculated with *M. loti* carrying dsRED and harvested at the indicated time after inoculation.

### Plasmid construction

All primers, oligonucleotides, and the synthetic DNA fragment are detailed in Supplementary Table 2.

For the overexpression analyses of *MIR2111-2*, *MIR2111-4*, and *MIR2111-5*, plasmids were constructed as follows. DNA fragments fully covering pri-miR2111 regions, as detected by RNA-seq were amplified from *L. japonicus* ecotype MG-20 genomic DNA by specific primer sets using the PrimeSTAR MAX DNA polymerase (Takara) and cloned into EcoRI-digested pENTR-1A (Invitrogen) using an In-Fusion HD Cloning Kit (Clontech). For hairy root transformation, all inserts were transferred to pUb-GW-GFP^47^ by LR clonase (Invitrogen). The insert of *MIR2111-5* fragment was also transferred to pUb-GW-Hyg^47^ by LR clonase for use in whole-plant transformation.

For *proUBQ:STTM2111* construct, the sequence of STTM2111 with attL1 and attL2 was synthesized (attL1:STTM2111:attL2) and cloned into pMA-RQ (GeneArt, Thermo Fisher Scientific). This fragment was cloned into pUb-GW-Hyg by LR clonase.

For the GUS reporter assay of *MIR2111-5*, a 3.0-kb DNA fragment upstream of *MIR2111-5* was amplified from *L. japonicus* ecotype MG-20 genomic DNA using specific primer sets by means of the PrimeSTAR MAX DNA polymerase (Takara) and cloned into EcoRI-digested pENTR-1A (Invitrogen) using an In-Fusion HD Cloning Kit (Clontech). The insert was transferred to pMDC162^55^ by LR clonase.

For CRISPR/Cas9 constructs of *MIR2111-5*, targeting sites were designed using the CRISPR-P 2.0 program^56^. Two gRNAs were used for each construct to delete a nearby miR2111a sequence in *MIR2111-5* on the *L. japonicus* genome. In order to create CRISPR/Cas9 constructs containing dual gRNA, we first modified the gRNA cloning vector pMR203 (provided by Dr. Mily Ron and Dr. Masaki Endo) as follows. DNA fragments of U6-26 promoter/BbsI/Chimera RNA (sgRNA) were amplified by two types of specific primers with multiple cloning sites (MCS) using the PrimeSTAR GXL DNA polymerase (Takara) from pMR203. Each DNA fragment was inserted between the ApaI and PstI sites of pMR203 to create new cloning vectors with different MCS, designated as pMR203_AB and pMR203_BC, which enable removal of a gRNA expression cassette after cloning of gRNA using standard restriction enzymes. The binary vector pMR285 (derived from pDe-Cas9)^57^ was also modified as follows. Two oligo DNAs, forward and reverse of pMR285_oligo (Supplementary Table 2), were annealed and inserted into the BbsI site of pMR203 to create MCS. The DNA fragment of this MCS was removed by PstI and inserted into the PstI site of pMR285 to create new binary vectors with MCS, designated as pMR285_AD. Then, oligonucleotide pairs for gRNA were annealed at 95 °C for 5 min and inserted into the BbsI site of pMR203_AB and pMR203_BC. The gRNA expression cassettes in pMR203_AB and pMR203_BC were removed using BamHI/SalI and SalI/XbaI, respectively. These fragments were inserted simultaneously between the BamHI and XbaI sites of pMR285_AD.

### Plant transformation

Whole-plant transformation of *L. japonicus* was performed using *Agrobacterium tumefaciens*-mediated method as described previously^58^. *A. tumefaciens* AGL1 strains carrying the plasmid of interest were cultured in LB liquid medium supplemented with appropriate antibiotics at 28 °C for 2 days. The seedlings of *L. japonicus* grown on the 0.9% agar medium containing B&D (24 °C dark for 3 days) were placed on sterilized filter papers (6 × 6 cm) impregnated with the prepared *A. tumefaciens* suspension and their hypocotyl were cut to ~3-mm segments. The hypocotyl segments were transferred onto 5-mm piles of sterilized filter papers (6 × 6 cm) supplemented with co-cultivation medium (1/10 Gamborg’s B5 salt mixture, 1/10 Gamborg’s vitamin solution, 0.5 µg mL^−1^ BAP, 0.05 µg mL^−1^ NAA, 5 mM MES (pH 5.2), 20 μg ml^−1^ acetosyringone, pH 5.5) and cultured for 3 days at 24 °C dark to facilitate the infection of *A. tumefaciens* to the hypocotyl segments. Then, the hypocotyl segments were transferred onto a callus induction medium (Gamborg’s B5 salt mixture, Gamborg’s vitamin solution, 2% sucrose, 0.2 µg mL^−1^ BAP, 0.05 μg mL^−1^ NAA, 10 mM (NH_4_)_2_SO_4_, 12.5 μg mL^−1^ meropenem, 20 μg mL^−1^ Hygromycin B, 0.6% agar, pH 5.5) and cultured for 2–5 weeks at 24 °C (16 h of light, 8 h of darkness). The hypocotyl segments were transferred onto a new callus induction medium every 5 days. The developed-calli were placed on the callus induction medium without Hygromycin B to induce primordia of adventitious shoot for 3–7 weeks at 24 °C (16 h light, 8 h dark). The calli were transferred onto a new medium every 5 days. When primordia of adventitious shoot became visible in calli, the calli were transferred onto a shoot elongation medium (Gamborg’s B5 salt mixture, Gamborg’s vitamin solution, 2% sucrose, 0.2 μg ml^−1^ BAP, 12.5 μg mL^−1^ meropenem, 0.6% agar, pH 5.5) and grown for 3–6 weeks at 24 °C (16 h of light, 8 h of darkness) to elongate shoot from leaf primordia. The calli were transferred onto a new callus induction medium every 7 days. The individual shoots from calli were detached and inserted into a root induction medium (1/2 Gamborg’s B5 salt mixture, 1/2 Gamborg’s vitamin solution, 1% sucrose, 0.5 μg ml^−1^ NAA, 0.9% agar, pH 5.5) and cultivated for 10 days at 24 °C (16 h of light, 8 h darkness). Then, they were inserted into root elongation medium (1/2 Gamborg’s B5 salt mixture, 1/2 Gamborg’s vitamin solution, 1% sucrose, 0.9% agar, pH 5.5) and grown until their root length became more than 3 cm. The generated transgenic plants were transplanted into vermiculite to obtain their seeds.

Hairy root transformation of *L. japonicus* roots was performed using *Agrobacterium rhizogenes*-mediated transformation, as described previously^58^. *A. rhizogenes* AR1193 strains carrying the plasmid of interest were grown on YEB medium containing 1.5% agar supplemented with appropriate antibiotics at 28 °C for 2 days. *A. rhizogenes* AR1193 strains were collected and suspended in 6 mL of sterilized water. The seedlings of *L. japonicus* were grown on a 0.9% agar medium containing B&D for a total of 3 days (darkness for 2 days and 16 h of light, 8 h of darkness for 1 day). The seedlings were placed in the *A. rhizogenes* suspension and cut in the middle of their hypocotyls. The seedlings without roots were placed on a co-cultivation medium for hairy root transformation (1/2 Gamborg’s B5 salt mixture, 0.01% sucrose, 0.9% agar, pH 5.5) for 3 days (24 °C darkness). Then, they were transferred onto vertical hairy root induction medium (Gamborg’s B5 salt mixture, Gamborg’s vitamin solution, 1% sucrose, 12.5 μg mL^−1^ meropenem, 0.9% agar, pH 5.5) and grown for 10 days at 24 °C (16 h of light and 8 h of darkness). The plants with transformed roots displaying fluorescence of a GFP transformation marker were selected and used for further analyses.

### GUS staining

Two-week-old *proMIR2111-5:GUS* introduced transgenic plants were incubated with ice-cold 90% acetone on ice for 10 min and then stained with GUS staining buffer (0.4 mg mL^−1^ X-Gluc, 50 mM phosphate buffer pH 7.0, 1 mM K_4_[Fe(CN)_6_], 1 mM K_3_[Fe(CN)_6_], and 0.1% Triton X-100) at 37 °C for 3 h. Stained plants were incubated at 37 °C for 1 h with acetic acid and ethanol (ratio 6:1) buffer to remove the chlorophyll background.

### Microscopic observation

An SZX16 stereomicroscope or a BX50 microscope (Olympus) was used to observe roots, nodules, and GUS-stained whole plants. Nodules with the neck at the basal region were counted in nodulation phenotype assays. For plastic sections, GUS-stained leaves, stems, or nodules were fixed with Formalin-Acetic-Alcohol buffer for 12 h at 4 °C and embedded in Technovit 7100 resin (Haraeus Kulzer). Sections were cut with a microtome RM2255 (Leica) at a thickness of 5 μm and counterstained with 0.1% safranin for 10 min at 55 °C.

### Library preparation and sequencing

The total RNA was extracted from true leaves of *L. japonicus* grown under the conditions described using PureLink Plant RNA Reagent (Thermo Fisher Scientific) and purified using RNeasy plant mini kit (Qiagen). The quality of RNA was evaluated using an Agilent 2100 Bioanalyzer with Agilent RNA 6000 Nano kit. RNA-seq libraries were prepared using NEBNext Ultra II RNA Library Prep Kit for Illumina (New England Biolabs) according to the manufacturer’s protocol. The prepared RNA-seq libraries were qualified by the Agilent 2100 Bioanalyzer using Agilent High Sensitivity DNA kit and quantified using a KAPA Library Quant Kit (Kapa Biosystems). Each library was diluted to 2 nM. Libraries were sequenced using NextSeq 550 (Illumina) and generated 81 bp single-end reads.

### Bioinformatic analysis

All acquired RNA-seq reads were qualified by FastQC (ver. 0.11.7) and adapter trimmed by Trimomatic^59^ (ver. 0.36, options: CROP:80 LEADING:30 TRAILING:30 SLIDINGWINDOW:4:15 HEADCROP:6 MINLEN:36). Trimmed reads were mapped to the *L. japonicus* genome (Gifu v1.2) using Hisat2^60^ (ver. 2.1.0). The mapped reads with a MAPQ score of <30 were filtered out using SAMtools^61^ (v1.9, options: view -bq 30) to handle only high mapping-quality scored reads for downstream analysis. The number of raw reads, trimmed reads, and mapped reads are shown in Supplementary Table 3. Filtered mapped reads were assembled and calculated TPM values using Stringtie^46^ (ver. 1.3.4d) with the default setting. To visualize read coverage in the Integrative Genomics Viewer^62^ (ver. 2.4.10), filtered mapped reads were normalized by bins per million mapped reads (BPM) using bamCoverage^63^ (ver. 3.3.1, options: --binSize=10 --normalizeUsing BPM --smoothLength 30).

In order to predict new miR2111 loci, we searched miR2111a, b, and c sequence from new *L. japonicus* genome assembly (Gifu v1.2) by BLASTn^41^. We identified a total of ten positions for miR2111a, miR2111b, or miR2111c from the *L. japonicus* genome. In all, 100 bp-upstream and -downstream sequences from the identified 21-bp miR2111 regions were extracted, and the secondary structure was predicted using the minimum free energy (MFE) algorithm of RNAstructure program^42^ (ver. 6.1). Next, we searched for pri-miR2111s expressing in *L. japonicus* using mapped our RNA-seq data. In order to detect pri-miR2111 expression from our RNA-seq data, RNA-Seq-based gene predictions were performed using Stringtie (ver. 1.3.4d) with the default setting. We then searched for pri-miR2111s from predicted potential *L. japonicus* genes based on our RNA-seq data by referring to the position of the miR2111 hairpin sequences, including those we predicted. Furthermore, we searched new potential miR2111 genes that were not detected in our RNA-seq data using BLASTn search of sequences for all miR2111 genes against the *L. japonicus* genome and identified one new potential miR2111 gene. Secondary structures of all new miR2111s were re-estimated using the full-length sequence of the predicted miR2111 genes using the MFE algorithm of RNAstructure (ver. 6.1). The genomic positions of miR2111s were visualized by R software (ver. 3.6.2) with chromoMap package^64^ (ver. 0.2).

### Phylogenetic analysis of miR2111 genes

Putative miR2111 genes were aligned with MAFFT^65^ (ver. 7.313) with default parameters. Aligned sequences were trimmed using trimAL^66^ (ver. 1.4.rev22, option: -automated1). Phylogenetic trees were constructed by maximum likelihood (ML) analysis with IQ-TREE^67^ (ver. 1.6.9). ML tree was visualized using R software (ver. 3.6.2) with ggtree package^68^ (ver. 1.14.6).

### qRT-PCR analysis

The total RNA including miRNA was extracted from either roots or true leaves of plants grown under the conditions described using NucleoSpine miRNA (MACHEREY-NAGEL Inc). For qRT-PCR of mRNA and miR2111 precursors, extracted RNA was reverse-transcribed using PrimeScript RT reagent Kit (Perfect Real Time) (Takara). Mature miR2111s were reverse-transcribed and adapter-ligated using the Mir-X miRNA First-Strand Synthesis Kit (Takara). Mature miR2111-specific primer and adapter-specific primer contained in Mir-X miRNA First-Strand Synthesis Kit were used for the amplification of mature miR2111s. qRT-PCR was carried out by LightCycler 96 (Roche Applied Science) using TB Green Advantage qPCR Premix (Takara). *Ubiquitin* and *ATP synthase* were used for normalization of expression levels. Primers of *Ubiquitin*^69^ and *ATP synthase*^12^ were synthesized as described previously. All primers are listed in Supplementary Table 2.

### Statistical analysis

Tukey’s honestly significant difference test (Tukey-HSD) was performed with R software (ver. 3.6.2). Two-sided Student’s *t* test was performed by Python (ver. 3.6.7) with SciPy library (ver. 1.1.0).

### Reporting summary

Further information on research design is available in the Nature Research Reporting Summary linked to this article.

## Supplementary information

Supplementary Information

Reporting Summary

## Data Availability

The raw RNA-seq reads have been deposited in the DDBJ Sequence Read Archive (DRA) under accession number DRA009878. Source data are provided with this paper.

## Source data

Source Data
