# Supplementary material for: MIR2111-5 locus and shoot-accumulated mature miR2111 systemically enhance nodulation depending on HAR1 in Lotus japonicus
Source: Nat Commun. 2020 Oct 15;11:5192. doi: 10.1038/s41467-020-19037-9 (PMC7562733; doi:10.1038/s41467-020-19037-9)
Supplement: Supplementary file 1 — Supplementary Information [file 41467_2020_19037_MOESM1_ESM.pdf]

Supplementary Information

***MIR2111-5* locus and shoot-accumulated mature miR2111 systemically enhance nodulation depending on HAR1 in *Lotus japonicus*.**

Nao Okuma, Takashi Soyano, Takuya Suzaki, Masayoshi Kawaguchi*

*Corresponding authors:
Masayoshi Kawaguchi
Tel: +81- 564-55-7564; Email address: masayosi@nibb.ac.jp

Supplementary Information file includes:
Supplementary Figures 1-6, Supplementary Tables 1-3

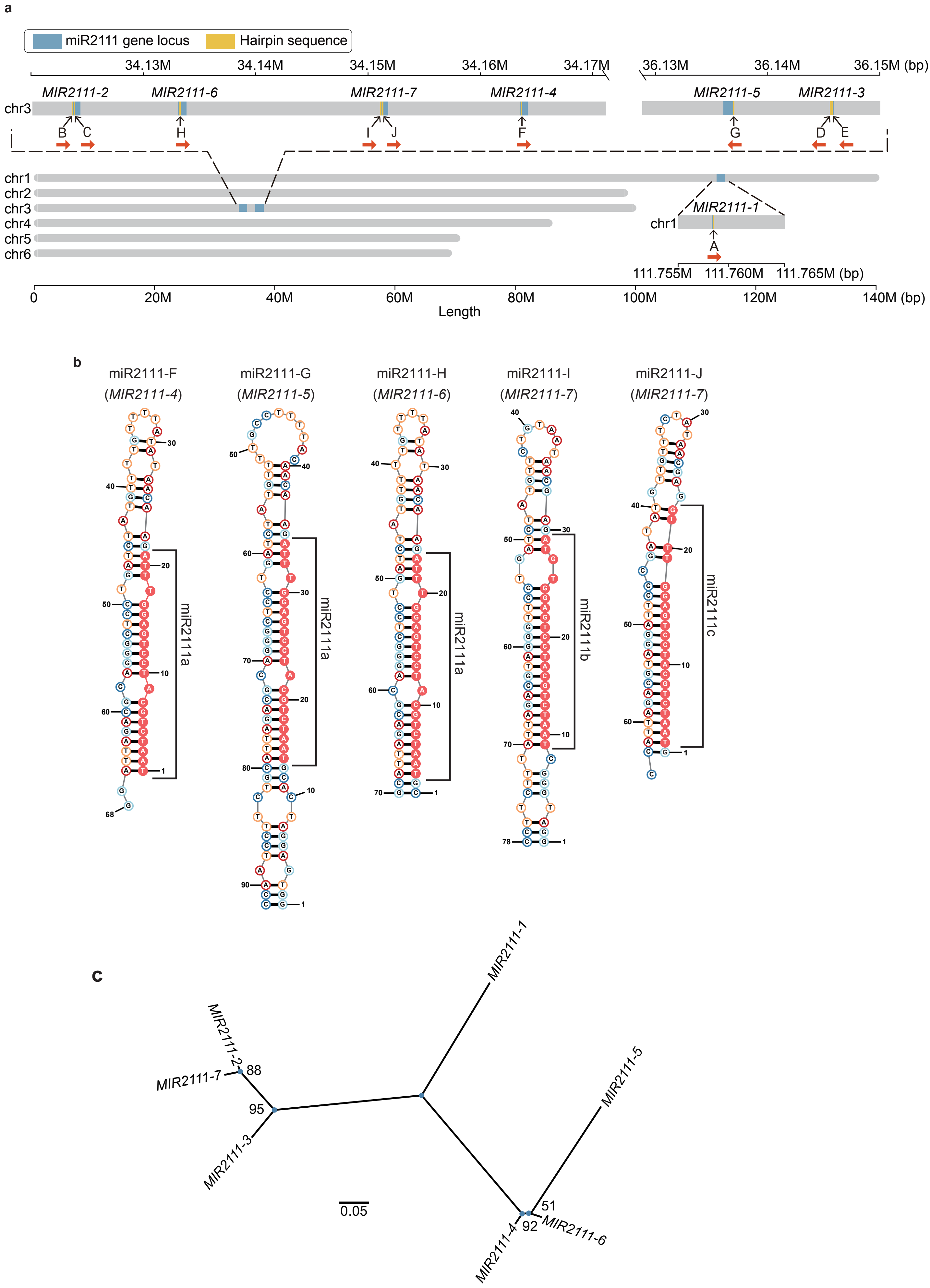
a
miR2111 gene locus
Hairpin sequence
34.13M
34.14M
34.15M
34.16M
34.17M
36.13M
36.14M
36.15M (bp)
MIR2111-2
MIR2111-6
MIR2111-7
MIR2111-4
MIR2111-5
MIR2111-3
chr3
B
C
H
I
J
F
G
D
E
chr1
chr2
chr3
chr4
chr5
chr6
MIR2111-1
chr1
A
111.755M
111.760M
111.765M (bp)
0
20M
40M
60M
80M
100M
120M
140M (bp)
Length
b
miR2111-F
(MIR2111-4)
miR2111-G
(MIR2111-5)
miR2111-H
(MIR2111-6)
miR2111-I
(MIR2111-7)
miR2111-J
(MIR2111-7)
miR2111a
miR2111a
miR2111a
miR2111b
miR2111c
c
MIR2111-1
MIR2111-2
88
MIR2111-7
95
MIR2111-3
MIR2111-5
51
92
MIR2111-4
MIR2111-6
0.05

Supplementary Figure 1:

Distribution of miR2111 genes on L. japonicus genome, secondary structures of news miR2111s, and phylogenetic relationship of miR2111 genes.

a Distribution of miR2111 genes on *L. japonicus* genome. Blue and yellow boxes indicate the positions of miR2111 genes (*MIR2111-1* to *MIR2111-7*) and miR2111 hairpin sequences (miR2111-A to miR2111-J), respectively. *MIR2111-2*, *MIR2111-3*, and *MIR2111-7* possess two miR2111 hairpin sequences. Red arrows represent the directions of mature miR2111s. **b** Hairpin structures of miR2111-F to miR2111-J. All secondary structures were predicted using the full-length sequence of the predicted miR2111 genes by means of the minimum free energy (MFE) algorithm of RNAstructure software (ver. 6.1). Nucleotides filled with red color indicate mature miR2111 sequences. **c** Maximum likelihood tree of miR2111 genes. Bootstrap values were calculated from 1000 times bootstrap replicates and described at each node. Scale bar represents the branch length.

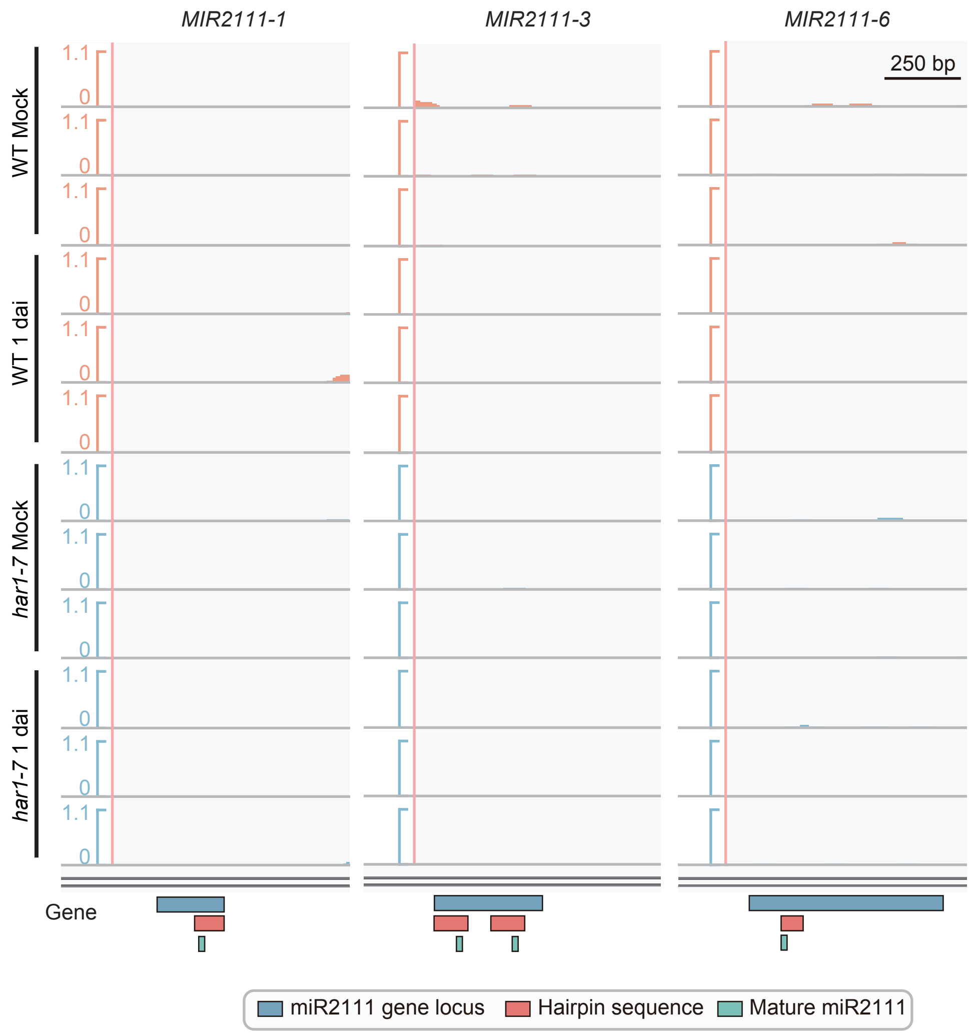

Supplementary Figure 2:

RNA-seq read coverage of *MIR2111-1*, *MIR2111-3*, and *MIR2111-6*.

RNA-seq read coverage of *MIR2111-1*, *MIR2111-3*, and *MIR2111-6*. All data were acquired by RNA-seq of mature leaves of wild-type (MG-20) and *har1-7* plants that were inoculated with *M. loti* (1 day after inoculation) or mock-treated (control). RNA-seq libraries were prepared with poly(A) enrichment methods. miR2111 genes were predicted by RNA-seq alignment assembly using Stringtie version 1.3.4d with default settings. Read abundance normalized in bins per million (BPM) is shown.

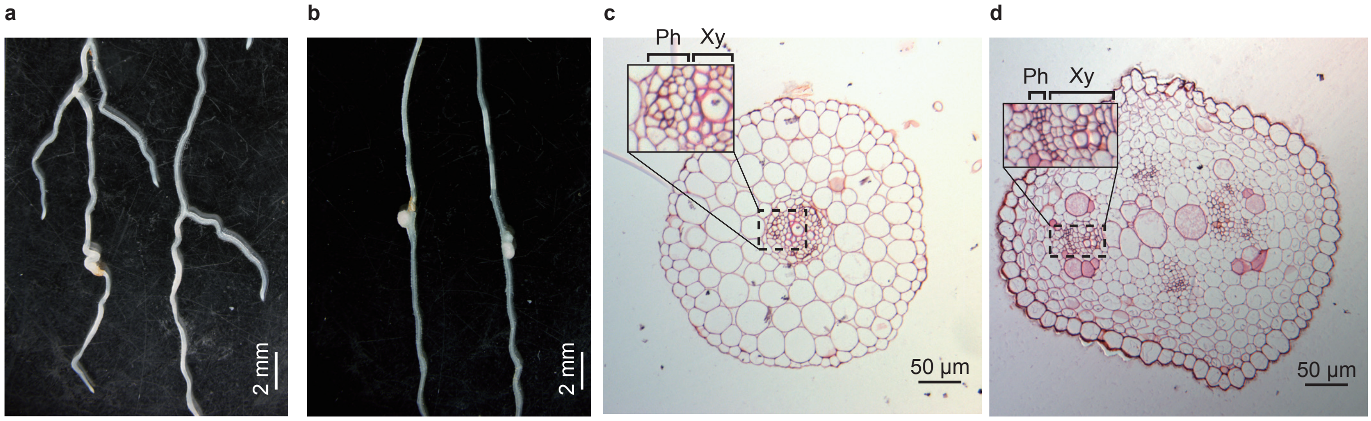

Supplementary Figure 3:

***MIR2111-5* was expressed neither in roots, root nodules, nor stems.**

a-d *L. japonicus* seedlings stably transformed with GUS reporter gene with expression driven by a 3.0 kb DNA fragment upstream of *MIR2111-5* were incubated in GUS staining buffer for 3 h. Roots (a, c), root nodules (7 days after inoculation) (b), and stems (d). Xy: xylem; Ph: phloem.

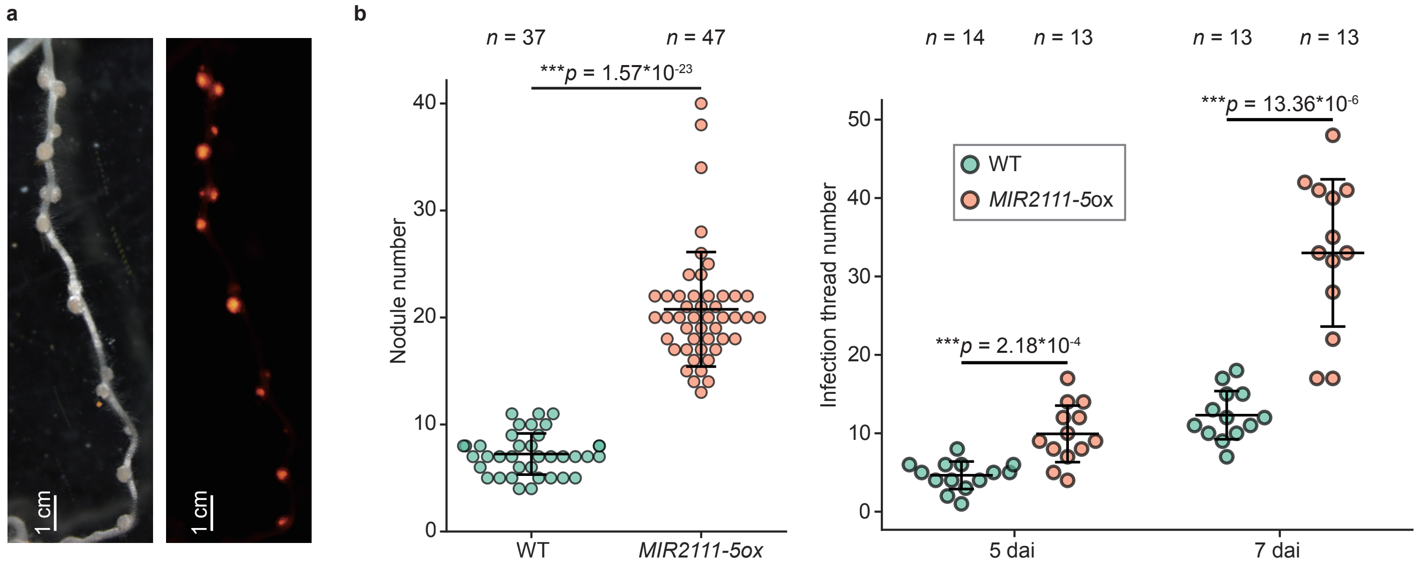

Supplementary Figure 4:

Over-expression of *MIR2111-5* induced hypernodulation and accumulation of mature miR2111s.

a Nodulation on *MIR2111-5*ox (21 days after inoculation). A bright image (Left) and corresponding fluorescence image of DsRed expressing in *M. loti* (Right). **b** Numbers of nodules (21 days after inoculation) and infection threads (5 and 7 days after inoculation) formed in wild-type and *MIR2111-5*ox roots. Scatterplots show individual biological replicates as dots. Bars indicate mean ± standard deviation. Two-sided Student’ s t-test was used to determine statistical difference compared with wild-type plants: ***, $p < 0.001$. Infection thread numbers of WT are from the same dataset presented in Fig. 5c since the experiments were conducted at the same time and conditions.

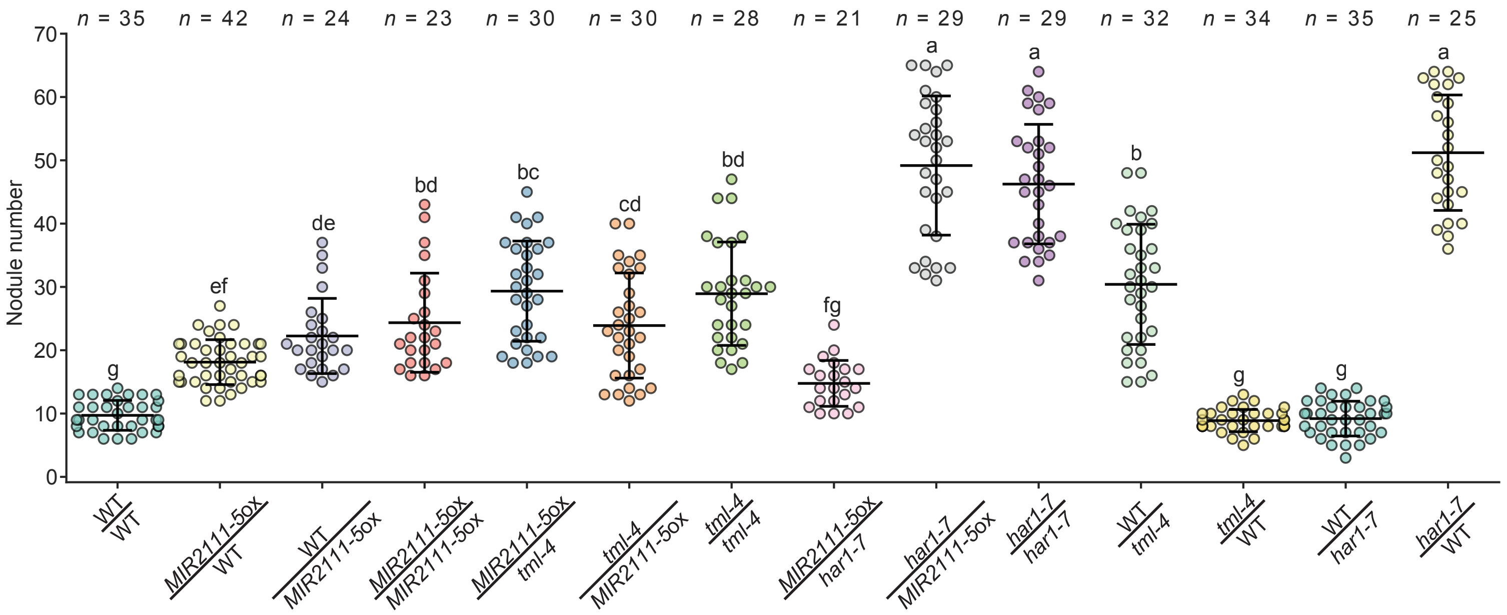

Supplementary Figure 5:

Reciprocal grafting of wild-type, *har1-7*, *tml-4*, and *MIR2111-5*ox.

Numbers of nodules (28 days after inoculation) formed in rootstocks of reciprocal- and self-grafted plants. Scatterplots show individual biological replicates as dots. Bars indicate mean ± standard deviation. Different letters indicate significant differences ($p < 0.05$) from Tukey’ s honestly significant difference test. Nodule number of WT/WT, *MIR2111-5*ox/WT, WT/*MIR2111-5*ox, and *MIR2111-5*ox/*MIR2111-5*ox are from the same dataset presented in Fig. 4e since the experiments were conducted at the same time and conditions.

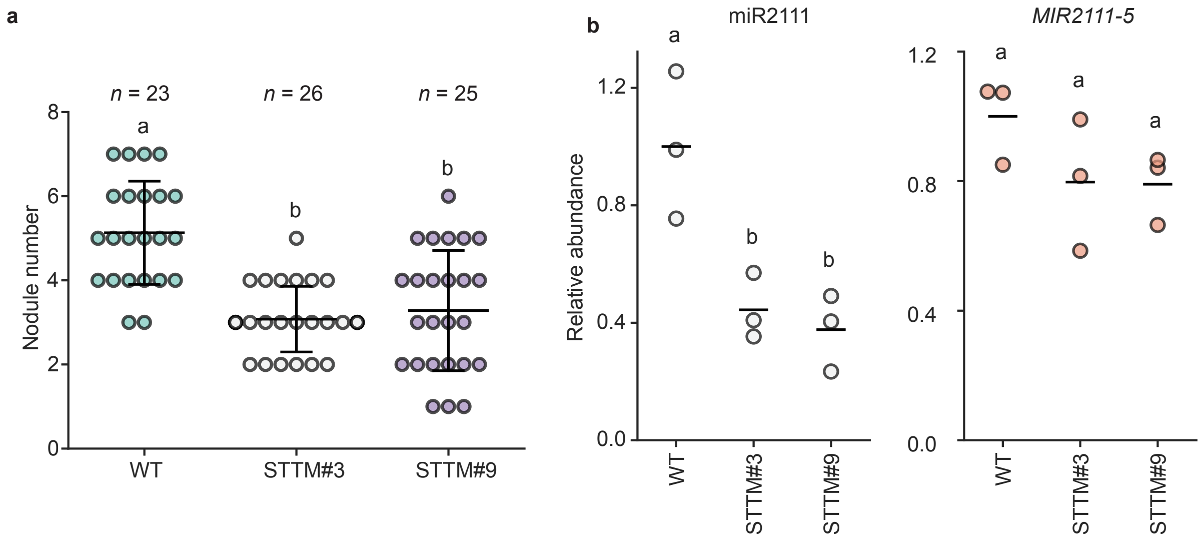

Supplementary Figure 6:

STTM2111 plants showed significant decrease in nodule numbers and mature miR2111s levels.

a Nodule numbers on wild-type and stable transformed lines of STTM2111 (21 days after inoculation). Bars indicate mean ± standard deviation. **b** qRT-PCR analyses of mature miR2111s and *MIR2111-5* in leaves of wild-type and STTM2111 lines. *n* = 3 individual biological replicates for each treatment. Bars indicate mean values. (a, b) STTM represents STTM2111 plants. Scatterplots show individual biological replicates as dots. Different letters indicate significant differences ($p < 0.05$) from Tukey’ s honestly significant difference test.

Supplementary Table 1: List of miR2111 genes, miR2111 hairpin sequences, and mature miR2111s.
miR2111 genes and corresponding miR2111 hairpin sequences and mature miR2111s are shown in table. The position and length columns refer to chromosomal positions and nucleotide sequence lengths of miR2111 genes and hairpin sequences, respectively.

miR2111 gene	Position (Gene)	Length (bp) (Gene)	Mature miR2111	Direction of mature miR2111	miR2111 hairpin sequence name	Position (Hairpin sequence)	Length (bp) (Hairpin sequence)	Reference
MIR2111-1	chr1:111758592-111758787	196	miR2111a	+	miR2111-A	chr1:111758703-111758787	85	Tsikou *et al*., 2018
MIR2111-2	chr3:34123742-34124530	789	miR2111b	+	miR2111-B	chr3:34123776-34123853	78	Tsikou *et al*., 2018
			miR2111c	+	miR2111-C	chr3:34123929-34124029	101	Tsikou *et al*., 2018
MIR2111-3	chr3:36146068-36145746	323	miR2111a	-	miR2111-D	chr3:36145845-36145747	99	Tsikou *et al*., 2018
			miR2111b	-	miR2111-E	chr3:36146011-36145914	98	Tsikou *et al*., 2018
MIR2111-4	chr3:34163935-34164541	607	miR2111a	+	miR2111-F	chr3:34164032-34164099	68	This study
MIR2111-5	chr3:36137160-36136263	898	miR2111a	-	miR2111-G	chr3:36137158-36137067	92	This study
MIR2111-6	chr3:34133426-34134047	622	miR2111a	+	miR2111-H	chr3:34133526-34133595	70	This study
MIR2111-7	chr3:34151024-34151700	677	miR2111b	+	miR2111-I	chr3:34151063-34151140	78	This study
			miR2111c	+	miR2111-J	chr3:34151231-34151294	64	This study

Supplementary Table 2: List of primers, oligonucleotides, and synthesized DNA fragment.

		Forward (5' --> 3')	Reverse (3' --> 5')
Cloning primers	*MIR2111-2*	ATCCGGTACCGAATTTCCATTGGCTCAATACTTGG	GTGCGGCCGCGAATTTCGGTCAAATTGACCAAAGC
	MIR2111-4	ATCCGGTACCGAATTTACAAGGACTCTTCTTATAGA	GTGCGGCCGCGAATTTTCAGGAAACTAGAACACAAA
	MIR2111-5	ATCCGGTACCGAATTGTTAGGTGAGGATCACGTA	GTGCGGCCGCGAATTAGGAAAACAAGGTTTAACCAT
	MIR2111-5 promoter	ATCCGGTACCGAATTGTTAGGGGCTAATCGATTTT	GTGCGGCCGCGAATTACTGATTCTTCCTTGTAGCT
qRT-PCR primers	miR2111	TAATCTGCATCCTGAGGTTTA	
	MIR2111-5	GGTTTAGAACAACATTTTCCG	GTAATCTGCGTCCCGAGGAC
	TML	GCCAACAATTGCCTGAAACCAGATG	CTTTATGGTGTTTCTCTCTATGAATGCTG
	Ubiquitin	ATGCAGATCTTCGTCAAGACCTTG	ACCTCCCCTCAGACGAAG
	ATP synthase	CAATGTCGCCAAGGCCCATGGTG	AACACCACTCTCGATCATTTCTCTG
Oligonucleotides for CRISPR gRNA	*mir2111-5-1A*	ATTGGAAGACGTAATCTGCGTCCCG	AAACCGGGACGCAGATTACGTCTTC
	mir2111-5-1B	ATTGGAAAATGTTGTTCTAAACCTC	AAACGAGGTTTAGAACAACATTTTC
	mir2111-5-2A	ATTGGCCCGAGGACTAGATACAAAA	AAACTTTTGTATCTAGTCCTCGGGC
	mir2111-5-2B	ATTGGCCTTGTAGCTTGTAGGACTG	AAACCAGTCCTACAAGCTACAAGGC
Primers and oligonucleotides for modification of pMR203 and pMR285	pMR203_AB	ATTGGGCCCTCTAGAGGATCCCCTAAGGTTGTACAAAAAAGC AGGCTTAC	ATTCTGCAGGGTACCGTCGACCCTAAGGTTTGTATAGAAA AGTTGGGTGC
	pMR203_BC	ATTGGGCCCGGATCCGTCGACCCTTAGGTTGTACAAAAAAGC AGGCTTAC	ATTCTGCAGGGTACCTCTAGACCTGAGGTTTGTATAGAAAA GTTGGGTGC
	pMR285_oligo	ATTGCTGCAGCCTAAGGAAAATCGGCCTGAGGGGATCCAATG CCGTCGACAATGCCTCTAGACCGATTGGTACCCTGCAG	AAACCTGCAGGGTACCAATCGGTCTAGAGGCATTGTCGAC GGCATTGGATCCCCTCAGGCCGATTTTCCTTAGGCTGCAG

Synthetic STTM miR2111-88 nt spacer with attL1 and attL2 (5' --> 3')	ATAGGGCGAATTGGCGGAAGGCCGTCAAGGCCGCATCAACGAGCTCCAAATAATGATTTTATTTTGACTGATAGTGACCTG TTCGTTGCAACAAATTGATAAGCAATGCTTTTTTATAATGCCAACTTTGTACAAAAAAGCAGGCTTAAACCTCAGGCTAATGC AGATTAGTTGTTGTTGTTATGGTCTAGTTGTTGTTGTTATGGTCTAATTTAAATATGGTCTAAAGAAGAAGAATATGGTCTAAA GAAGAAGAATTAAACCTCAGGCTAATGCAGATTAACCCAGCTTTCTTGTACAAAGTTGGCATTATAAGAAAGCATTGCTTATC AATTTGTTGCAACGAACAGGTCACTATCAGTCAAAATAAAATCATTATTTGGGTACCCTCTGGGCCTCATGGGCCTTCCGCT CACTGCCCGCTTTCC

Supplementary Table 3: Summary of RNA-seq reads.

Index name	Description	No. of raw reads	No. of trimmed reads	No. of mapped reads (more than MAPQ = 30)
idx1	WT (MG-20) Mock replicate 1	30,808,995	30,467,270	28,445,920
idx2	WT (MG-20) Mock replicate 2	34,663,575	34,302,126	31,964,562
idx3	WT (MG-20) Mock replicate 3	26,253,097	25,932,116	24,084,516
idx4	WT (MG-20) inoculated replicate 1	30,185,644	29,859,787	27,719,358
idx5	WT (MG-20) inoculated replicate 2	27,191,690	26,905,799	24,975,835
idx6	WT (MG-20) inoculated replicate 3	27,041,726	26,749,774	24,824,295
idx10	har1-7 Mock replicate 1	25,996,589	25,737,531	23,537,190
idx11	har1-7 Mock replicate 2	25,387,350	25,113,274	22,774,526
idx12	har1-7 Mock replicate 3	26,440,567	26,174,251	24,189,348
idx13	har1-7 inoculated replicate 1	25,124,702	24,845,951	23,065,335
idx14	har1-7 inoculated replicate 2	22,991,791	22,749,322	21,146,191
idx15	har1-7 inoculated replicate 3	35,760,799	35,378,641	32,912,653
